# Translational pathology in drug discovery

**DOI:** 10.3389/fphar.2024.1409092

**Published:** 2024-06-10

**Authors:** Snježana Čužić, Maja Antolić, Anja Ognjenović, Vuk Milutinović, Sonja Vidović Iviš, Ines Glojnarić, Martina Bosnar, Lidija Požgaj, Ema Prenc, Vesna Eraković Haber

**Affiliations:** ^1^ In vivo Pharmacology and Toxicology, Selvita, Zagreb, Croatia; ^2^ In vitro Pharmacology, Selvita, Zagreb, Croatia; ^3^ Pharmacology and Translational Research, Selvita, Zagreb, Croatia

**Keywords:** translational medicine, translational pathology, drug discovery, animal models, *in vitro*

Throughout its history, humanity has been exposed to various diseases. As human society evolved, the incidence and prevalence of communicable and non-communicable diseases varied over centuries. Attempting to influence the disease course and prevent mortality, societies have given much attention to inventing new treatment methods and developing drugs. As a result of those efforts, drug discovery expenditures have been constantly increasing in the past years, and it seems that they will keep increasing over the years to come. Although, during the preclinical phase of drug discovery, great efforts are put into the selection of the best molecules for further progression (Dowden and Munro, 2019), a lack of efficacy in humans contributes significantly to a high attrition rate of new molecular entities in the clinical trials (Schlander et al., 2021; Sun et al., 2022) and calls for innovative scientific solutions in pharmaceutical research and development.

Translational medicine (TM) is a rather recent, but rapidly developing scientific field, defined by the European Society for Translational Medicine as an “interdisciplinary branch of the biomedical field supported by three main pillars: benchside, bedside, and community. The goal of TM is to combine disciplines, resources, expertise, and techniques within these pillars to promote enhancements in prevention, diagnosis, and therapies” (Cohrs et al., 2015). Translational medical research is divided into five stages: T0 research represents basic *in vitro* and *in vivo* research; T1 research stage covers the translation of basic research knowledge to humans (target engagement, Phase 1 clinical research) followed by T2 stage investigating success of translation to patients (Phase 2 and 3 clinical trials) while in the T3 research stage success of clinical implementation is evaluated, and finally, translation to communities is in the focus of T4 research stage (Callard et al., 2011; IOM, 2013).

**FIGURE 1:**
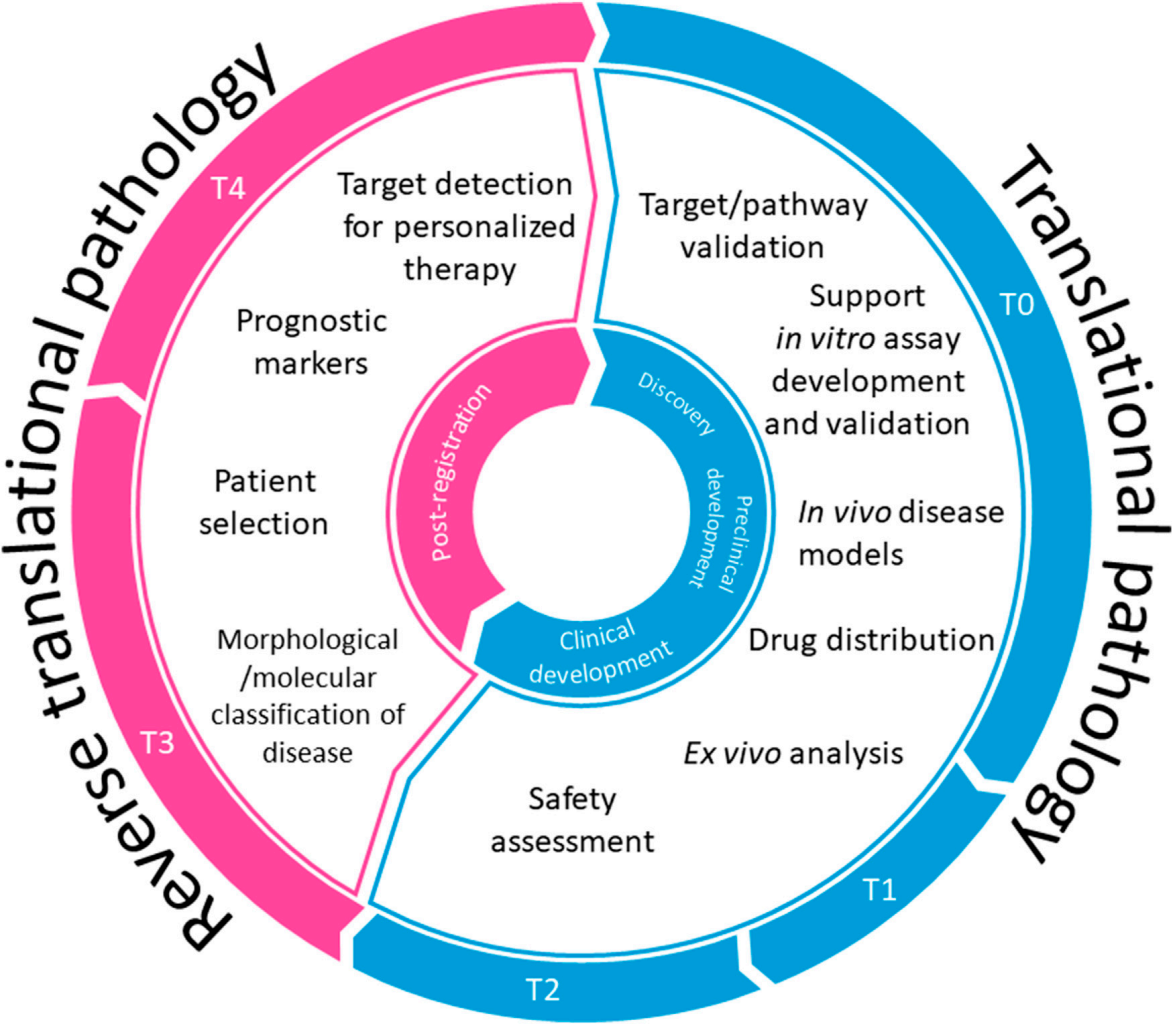
Translational pathology in drug discovery.

Translational pathology has been opening the doors for research within translational medicine by translating clinical data into basic research. Therefore, pathologists have been encouraged to participate in “reverse translational research” that broadens the knowledge of mechanisms underlying known clinical entities (Zhang, 2022). On the other hand, under the umbrella of translational research, translation pathology focuses on applying the knowledge gained by basic science research to clinical practice (Translational Science Spectrum, 2020). In the eyes of the authors, translational pathology may be regarded as an even broader scientific discipline, offering opportunities to improve the drug discovery process by helping to circumvent numerous obstacles, paving the path from target validation to clinical trials and reverse, from clinical to basic research (Figure 1), thus contributing to building up a bridge over “Translational gap” (Hartl et al., 2021) and “Walley of death” (Seyhan, 2019). We will try to outline the possible use of data gained by pathohistological evaluation along the drug discovery process highlighting target validation, design of an *in vitro* and *in vivo* screening cascade, pharmacokinetic studies, and clinical trials including the impact of reverse translational pathology on drug discovery process.

Target validation is a crucial step in the early, Stage 1 drug discovery process (Singh et al., 2023). Possible drug targets are identified in various biological studies, ranging from molecular biology interventions to clinical research. The usage of knock-in and knock-out experimental animals and cell lines enabled a detailed study of tissue changes at molecular and morphological levels, induced by gene/protein alterations, important to understand human disease pathology (Doyle et al., 2012). Nevertheless, not all suggested drug targets, proven to play a role in disease-related *in vitro* systems and/or animal models, have had an impact on human disease course. Numerous strategies have been implemented to reduce the drug attrition rate caused by insufficient drug target validation including AstraZeneca’s “5R framework” strategy (Morgan et al., 2018) that embraces the “Right target within the right tissue in the right patient” motto. Although such strategies have improved the success rate in drug development, efficient target assessment remains a difficult task (Emmerich et al., 2021).

**FIGURE 2:**
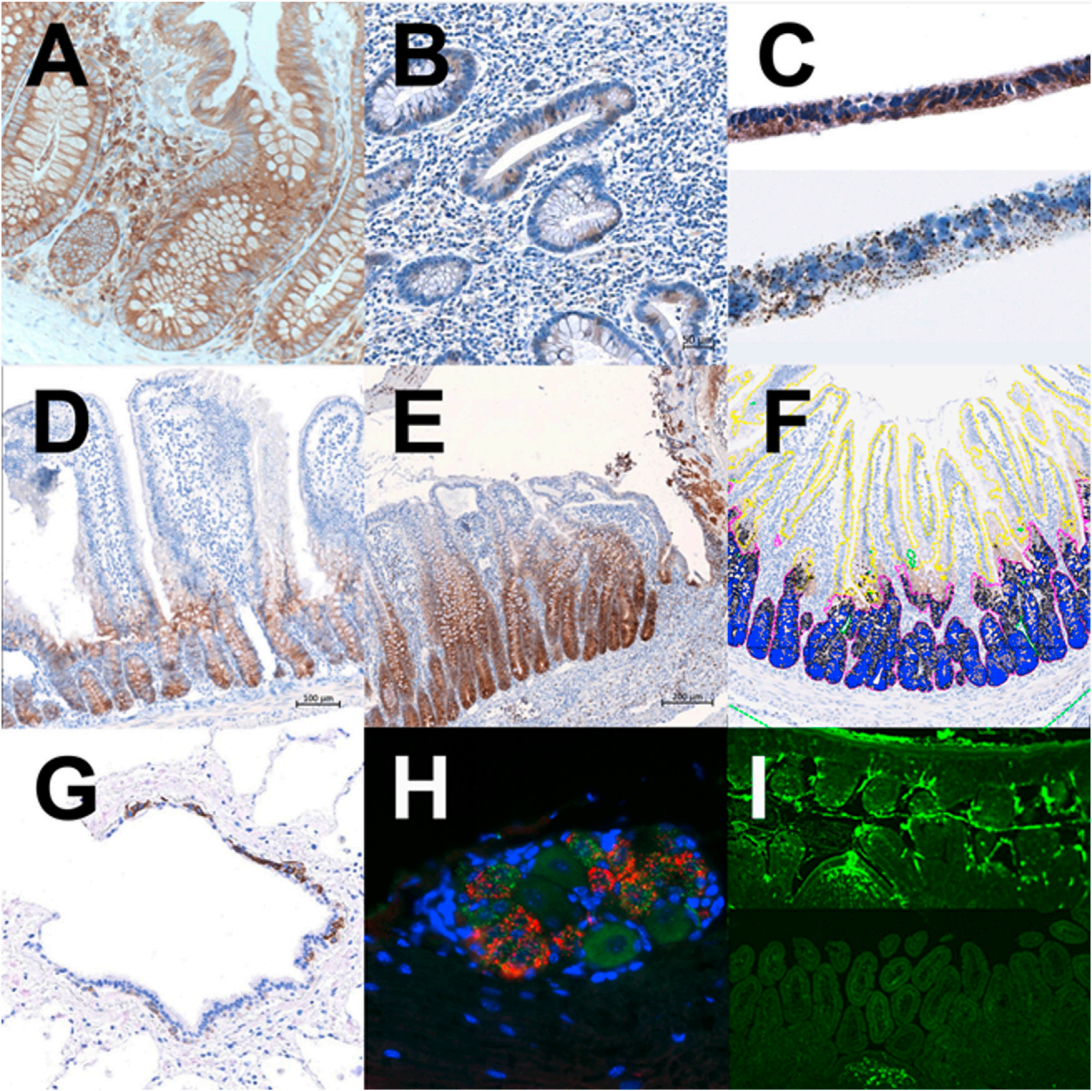
Translational pathology in the drug discovery process. **(A)** Target expression in non-IBD human colon mucosa, IHC. **(B)** Target expression in IBD human colon mucosa, IHC. **(C)** Target expression in cell line CaCo2 grown on a membrane in vitro, IHC/ISH. **(D)** Target expression in naïve rat small intestine, IHC. **(E)** Target expression in the model of indomethacin-induced mucosa damage in rat small intestine, IHC. **(F)** Digital image analysis of target expression in stem-cell niche vs villus epithelium, Visiopharm software. **(G)** Visualization of epithelial cell subtype in human lungs; Precision-cut lung slices, IHC. **(H)** Target validation in murine dorsal root ganglion, double ISH. **(I)** Tracking the labeled oligonucleotide within the murine intestine at 3 hours and 24 hours post per os application; IF.

Translational pathology may support efforts to achieve the above-defined 5R research goal by studying drug-target expression dynamics, on a protein and mRNA level, in human disease along its’ developmental path and by comparing target expression during various stages of the disease to the pattern within non-diseased tissue (Figures 2A, B). In contrast to methods based on the analysis of tissue homogenates, the evaluation of histological slides offers insight into target-bearing cell type (Čužić et al., 2021), as well as cell-cell, cell-extracellular matrix interactions within the tissue (Zidar et al., 2020; Mahdi et al., 2023), and impact of active substances secreted by constitutive, metaplastic (Antolić et al., 2019) or infiltrating cells, thus enabling understanding of physiological and pathophysiological processes taking place in their natural environment. Through decades of research, it became evident, that the cellular “environment” plays a crucial role in the cell phenotype through its’ life; during development, in health, and in disease. Understanding the importance of the environment and structural organization of tissue and its’ niches, gained through thorough pathological evaluation, initiated the development of an array of new omics and spatial technologies that are being employed to study the pathophysiology of human disease within the spatial context (Conrad and Altmüller, 2022; Britt, 2023). Further, a significant effort has been invested to develop *in vitro* system(s) representing human tissue structure and function, providing experimental conditions as close as possible to human tissue/disease environment (Allen et al., 2023).

The design of an *in vitro* and *in vivo* screening cascade focused on the targeted patient population relies on a deep understanding of the disease and comprehensive translational research. Research within the field of translational pathology notably contributes to the elucidation of possible impediments and enables the bypassing of drawbacks along the drug discovery path. A dataset gained during the target/pathway validation phase of the early drug research process is essential to set up a novel drug/antibody/oligonucleotide selection cascade and optimal biomarkers that should be monitored throughout the whole drug discovery and development process. One of the crucial steps during early compound efficacy testing is the establishment of *in vitro* assays, either using cell lines, primary human/rodent cells, or human tissue. Pathohistological evaluation could contribute to translating data collected from human disease into *in vitro* systems that should closely outline the pathophysiological process of interest by determining the level of drug-target expression (Figures 2A, B, D, E–G), target-synthesis at mRNA level (Figure 2H), targets’ (sub) cellular location within the tissue (Bosnar et al., 2011) and cell culture at various time-points during *in vitro* cultivation period (Figure 2C). Histological readouts on downstream events, upon biologically relevant trigger and/or pharmacological treatment in the *in vitro* culture of tissue explants from healthy and diseased donors, naïve and genetically modified animals, animal models, and/or 3D cell cultures, could highlight differences in physiological and pathophysiological pathways among species and experimental conditions used for novel drug selection. It is the opinion of the authors that a comprehensive understanding of experimental *in vivo* models is of uttermost importance for obtaining data relevant to human disease. Understanding the extent of translatability, including limitations, of animal models to human disease, is important for decision-making in the drug discovery process. The pathohistological analysis could provide powerful insight (Čužić et al., 2021). It may prove valuable to investigate the presence and function of multiple target-expressing cell types (epithelial/mesenchymal cell types, resident/infiltrating inflammatory cells, metaplastic, dysplastic or tumor cells, etc.) in naïve tissue, human disease, and experimental models. At the same time, drug targets usually are not expressed by only one cell type. Further, the target expression in laboratory animals does not always reflect target expression in humans, varies among rodent (murine/rat) strains, and expression dynamics in animal models do not necessarily correspond to the human disease itself (Čužić et al., 2021). Investigating cells’ life-cycle and functional circuits of (immunological) cells at different stages of the disease additionally could shed light on the intricate disease development and multiple organ responses to the drug intervention and could prove important in the clinical research phase. On the other hand, the pathological examination has revealed great heterogeneity of tissue composition and spatial distribution of different cell (sub)types within tumor samples from different patients (Massa and Seliger, 2023) with likely implications on treatment outcomes. This calls for another level of complexity in setting up appropriate *in vitro* and *in vivo* testing systems for oncology drug development.

Although pathologic evaluation is not frequently included in pharmacokinetic studies during preclinical research, there is a possibility to capture the distribution and accumulation of labeled drugs/oligonucleotide within organs, tissues, and cell types *in vivo* (Figure 2I) (Matijašić, 2012), is more and more addressed by label-free methods combining histology and mass spectrometry (Spruill, 2022). Such evaluation provides a true basis for PK/PD assessment that considers drug distribution not only among different organs but also different tissues and different cells within tissues.

Throughout the entire translational circle, starting with patients, followed by experimental and toxicological studies, and ending with clinical trials, pathohistological analysis proved to contribute to an overall understanding of a disease and therapy outcome (beneficial and/or adverse). Multiple drug targets have been brought to light by the reverse translational pathology studies of their expression and potential role in human disease, αV integrin chain being only one illustration among of many examples. The first integrins were described in 80ties by a group of Erkki Ruoslahti (Pytela et al., 1985; Gehlsenlena et al., 1988). Soon followed pathohistological reports on their expression in kidneys (Čužić, 1991; Waldherr et al., 1992; Shimizu et al., 2006) and their potential role in glomerulonephritis. The expression of αV, β1, and β3 integrin chains within crescents in the extraglomerular proliferative form of glomerulonephritis was brought to light (Baraldi A. ate al, 1995) and confirmed by the following studies (Sonnenberg et al., 1990; Hillis et al., 1997; Roy-Chaudhury et al., 1997; Kagami et al., 2004). Decades later, it has been shown that pharmacological inhibition of αvβ1 integrin in experimental settings ameliorates renal failure (Chang et al., 2017). Experimental investigation of αVβ1, αVβ3, and αVβ6 integrin role in *vitro* (Andjus et al., 2018) and in animal models covering a broader span of conditions like fibrosis (Henderson and Sheppard, 2013) and cancer (Hamidi and Ivaska, 2018) identified integrins as a possible drug-targets. Based on data gathered along the challenging path of pre-clinical research, new chemical entities designed for defined therapy areas are currently tested in humans; αVβ1, αVβ3 and αVβ6 integrin inhibitors for the treatment of glioblastoma (Cilengitide) (Stupp et al., 2014) and pulmonary fibrosis (PLN-74809) (Pliant Therapeutics https://clinicaltrials.gov/ct2/show/NCT04396756) found their way to the clinic.

Last but not least, pathological classifications of diseases, as well as novel molecular pathology approaches, that cluster patients into subgroups likely to respond to therapeutic intervention, provide the basis for progress toward a personalized medicine approach. This has been most prominent in the field of oncology, as shown in the example of mamma carcinoma (Zhang, 2023) and GIST tumor (Mechahougui et al., 2023).

In conclusion, introducing translational pathology, as an ineluctable element to a drug-discovery process, in the opinion of the authors, ensures an understanding of drug-target physiology and its’ role in disease-related pathophysiology, the importance of which has been underlined by GOT-IT (Guidelines on Target Assessment for Innovative Therapeutics) recommendations and summarized within critical path questions defined by the authors (Emmerich et al., 2021).
